# [*N*-Benzyl-*N*-(diphenyl­phosphanylmeth­yl)pyridin-2-amine]­chloridomethyl­platinum(II)

**DOI:** 10.1107/S1600536810049652

**Published:** 2010-12-04

**Authors:** Zhi-Wei Wang, Peng Qiao, Zi-Jia Wang, Chong-Qing Wan

**Affiliations:** a Department of Chemistry, Capital Normal University, Beijing 100048, People’s Republic of China

## Abstract

In the mononuclear title complex, [Pt(CH_3_)Cl(C_25_H_23_N_2_P)], the *N*-benzyl-*N*-(diphenyl­phosphanylmeth­yl)pyridin-2-amine functions as a bidentate ligand with the pyridyl N atom and the phosphine P atom chelating the Pt^II^ ion, forming a six-membered metallocycle. The Pt^II^ atom adopts a square-planar coordination geometry with one methyl group and one chloride ligand bonding to the metal center in a *cis* relationship. C—H⋯π and C—H⋯Cl inter­actions help to consolidate the packing.

## Related literature

For coordination complexes with hemilabile ligands with PN donor sets, see: Espinet & Soulantica (1999 ▶); Song *et al.* (2001 ▶); Wang *et al.* (2010 ▶). For coordination complexes with the *N*-benzyl-*N*-(diphenyl­phosphanylmeth­yl)pyridin-2-amine ligand, see: Li *et al.* (2003 ▶, 2006 ▶). For hydrogen bonds, see: Desiraju & Steiner (1999 ▶) and for C—H⋯π inter­actions, see: Umezawa *et al.* (1998 ▶).
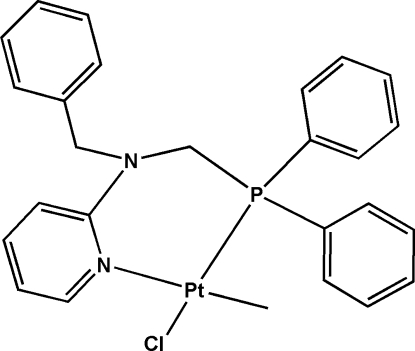


## Experimental

### 

#### Crystal data

[Pt(CH_3_)Cl(C_25_H_23_N_2_P)]
*M* *_r_* = 628.00
Triclinic, 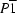

*a* = 9.538 (3) Å
*b* = 10.770 (3) Å
*c* = 13.125 (4) Å
α = 98.367 (4)°
β = 106.256 (4)°
γ = 107.266 (5)°
*V* = 1197.0 (6) Å^3^
*Z* = 2
Mo *K*α radiation
μ = 6.06 mm^−1^
*T* = 293 K
0.33 × 0.24 × 0.20 mm

#### Data collection

Bruker APEXII CCD area-detector diffractometer
Absorption correction: multi-scan (*SADABS*; Bruker, 2007 ▶) *T*_min_ = 0.675, *T*_max_ = 1.000
6265 measured reflections
4201 independent reflections
3270 reflections with *I* > 2σ(*I*)
*R*_int_ = 0.041

#### Refinement

*R*[*F* ^2^ > 2σ(*F* ^2^)] = 0.050
*wR*(*F* ^2^) = 0.123
*S* = 1.01
4201 reflections
281 parameters
H-atom parameters constrained
Δρ_max_ = 2.18 e Å^−3^
Δρ_min_ = −2.33 e Å^−3^

### 

Data collection: *APEX2* (Bruker, 2007 ▶); cell refinement: *APEX2* and *SAINT* (Bruker, 2007 ▶); data reduction: *SAINT*; program(s) used to solve structure: *SHELXS97* (Sheldrick, 2008 ▶); program(s) used to refine structure: *SHELXL97* (Sheldrick, 2008 ▶); molecular graphics: *SHELXTL* (Sheldrick, 2008 ▶); software used to prepare material for publication: *SHELXTL* and *PLATON* (Spek, 2009 ▶).

## Supplementary Material

Crystal structure: contains datablocks I, global. DOI: 10.1107/S1600536810049652/zq2076sup1.cif

Structure factors: contains datablocks I. DOI: 10.1107/S1600536810049652/zq2076Isup2.hkl

Additional supplementary materials:  crystallographic information; 3D view; checkCIF report

## Figures and Tables

**Table 1:** Hydrogen-bond geometry (Å, °) *Cg*1 is the centroid of the C15–C20 benzene ring.

*D*—H⋯*A*	*D*—H	H⋯*A*	*D*⋯*A*	*D*—H⋯*A*
C23—H23⋯*Cg*1^i^	0.93	3.10	4.001 (2)	154
C7—H7*B*⋯Cl1^ii^	0.97	2.72	3.392 (2)	127

Symmetry codes: (i) 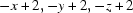; (ii) 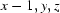.

**Fig. 1.**
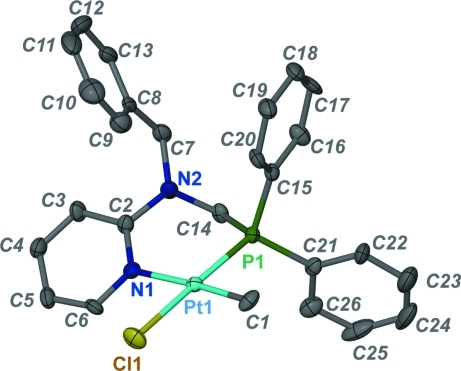
The atom-numbering scheme of the title complex. Displacement ellipsoids are drawn at the 30% probability level and H atoms are omitted for clarity.

**Fig. 2.**
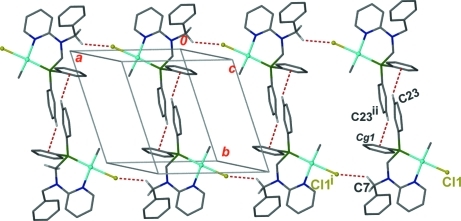
The C—H(benzene)···π and C—H(methylene)···Cl interactions between the mononuclear units arranged along the a direction. The Cg1 represents the centroid of the C15—C16—C17—C18—C19—C20 ring (benzene). Symmetry codes: (i) x + 1, y, z; (ii) -x + 2, -y + 2, -z + 2.

## supplementary crystallographic information

### Comment

Rigid hemilabile ligands such as (2-diphenylphosphanyl)pyridine (Espinet *et al.*, 1999) and 2,6-bis(diphenylphosphanyl)pyridine (Song *et al.*, 2001; Wang *et al.*, 2010) have extensively been studied, including the fascinating structures and the catalytic properties of their metal complexes. We here report a new complex with the *N*-benzyl-*N*-(diphenylphosphanylmethyl)pyridin-2-amine ligand (abbreviated as *L*), namely Pt(*L*)MeCl.

In the title complex, the pyridyl N atom and the phosphine P atom bond to the square-planar coordinated Pt^II^ ion in relative *cis* sites, generating a six-membered ring similar to the reported Re(*L*)(CO)_3_Cl (Li *et al.*, 2006), as shown in Fig. 1. In the crystal structure of the title complex, the main intermolecular non-covalent interactions are C—H(benzene)···π (Umezawa *et al.*, 1998) and C—H(methylene)···Cl (Desiraju & Steiner, 1999) interactions, which connect the mononuclear units along the *a* direction to form a chain structure. As shown in Fig. 2, the C23^ii^—H23^ii^···*Cg*1 contact exhibits a C23···*Cg*1 distance of 4.001 (2) Å and a C23—H23···*Cg*1 bond angle of 154° (*Cg*1 represents the centroid of the C15—C16—C17—C18—C19—C20 benzene ring [symmetry code: (ii) -*x* + 2, -*y* + 2, -*z* + 2]. Regarding the C7—H7B(methylene)···Cl1^i^ contact, the C7···Cl1^i^ distance is 3.392 (2) Å and the C7—H7B···Cl1^i^ angle equals 127° [symmetry code: (i) *x* + 1, *y*, *z*].

### Experimental

*N*-benzyl-*N*-(diphenylphosphanylmethyl)pyridin-2-amine (*L*) was obtained by the reaction of *N*-benzylpyridin-2-amine with Ph_2_PH and (HCHO)*_n_*, using a developed method of the Mannich reaction in acidic medium as reported in literature (Li *et al.*, 2003). A mixture of Pt(COD)MeCl (0.210 g, 0.6 mmol) and 0.382 g of *L* (1.0 mmol) in CH_2_Cl_2_ (20 ml) was stirred at room temperature for 3 h (COD = 1,5-cyclooctadiene). The clear solution was filtered and the solvent was concentrated to a small volume and diethyl ether was subsequently added to give 0.309 g (82%) of the title complex as a white solid, crystals of which were obtained after four days by recrystallization from CH_2_Cl_2_/*n*-hexane, yield: 0.230 g (61%).

### Refinement

The hydrogen atoms were placed in idealized positions and allowed to ride on the relevant carbon atoms, with C—H = 0.93 Å and *U*_iso_ = 1.2*U*_eq_(C) for aromatic H atoms, with C—H = 0.97 Å and *U*_iso_ = 1.2*U*_eq_(C) for methylene H atoms, and with C—H = 0.96 Å and *U*_iso_ = 1.5*U*_eq_(C) for methyl H atoms.

### Crystal data

**Table:**

[Pt(CH_3_)Cl(C_25_H_23_N_2_P)]	*V* = 1197.0 (6) Å^3^
*M**_r_* = 628.00	*Z* = 2
Triclinic, *P*1	*F*(000) = 612
Hall symbol: -P 1	*D*_x_ = 1.742 Mg m^−^^3^
*a* = 9.538 (3) Å	Mo *K*α radiation, λ = 0.71073 Å
*b* = 10.770 (3) Å	µ = 6.06 mm^−^^1^
*c* = 13.125 (4) Å	*T* = 293 K
α = 98.367 (4)°	Block, colourless
β = 106.256 (4)°	0.33 × 0.24 × 0.20 mm
γ = 107.266 (5)°	

### Data collection

**Table:**

Bruker APEXII CCD area-detector diffractometer	4201 independent reflections
Radiation source: fine-focus sealed tube	3270 reflections with *I* > 2σ(*I*)
graphite	*R*_int_ = 0.041
ω scans	θ_max_ = 25.0°, θ_min_ = 2.3°
Absorption correction: multi-scan (*SADABS*; Bruker, 2007)	*h* = −11→6
*T*_min_ = 0.675, *T*_max_ = 1.000	*k* = −12→12
6265 measured reflections	*l* = −15→15

### Refinement

**Table:**

Refinement on *F*^2^	Primary atom site location: structure-invariant direct methods
Least-squares matrix: full	Secondary atom site location: difference Fourier map
*R*[*F*^2^ > 2σ(*F*^2^)] = 0.050	Hydrogen site location: inferred from neighbouring sites
*wR*(*F*^2^) = 0.123	H-atom parameters constrained
*S* = 1.01	*w* = 1/[σ^2^(*F*_o_^2^) + (0.065*P*)^2^] *P* = (*F*_o_^2^ + 2*F*_c_^2^)/3
4201 reflections	(Δ/σ)_max_ = 0.001
281 parameters	Δρ_max_ = 2.18 e Å^−^^3^
0 restraints	Δρ_min_ = −2.33 e Å^−^^3^

### Special details

**Table:**

Geometry. All e.s.d.'s (except the e.s.d. in the dihedral angle between two l.s. planes) are estimated using the full covariance matrix. The cell e.s.d.'s are taken into account individually in the estimation of e.s.d.'s in distances, angles and torsion angles; correlations between e.s.d.'s in cell parameters are only used when they are defined by crystal symmetry. An approximate (isotropic) treatment of cell e.s.d.'s is used for estimating e.s.d.'s involving l.s. planes.
Refinement. Refinement of *F*^2^ against ALL reflections. The weighted *R*-factor *wR* and goodness of fit *S* are based on *F*^2^, conventional *R*-factors *R* are based on *F*, with *F* set to zero for negative *F*^2^. The threshold expression of *F*^2^ > σ(*F*^2^) is used only for calculating *R*-factors(gt) *etc*. and is not relevant to the choice of reflections for refinement. *R*-factors based on *F*^2^ are statistically about twice as large as those based on *F*, and *R*- factors based on ALL data will be even larger.

### Fractional atomic coordinates and isotropic or equivalent isotropic displacement parameters (Å^2^)

**Table:**

	*x*	*y*	*z*	*U*_iso_*/*U*_eq_	
Pt1	0.99999 (4)	0.54883 (4)	0.70540 (3)	0.03520 (15)	
Cl1	1.1825 (3)	0.4495 (3)	0.6820 (3)	0.0641 (8)	
P1	0.8409 (3)	0.6473 (2)	0.73503 (19)	0.0340 (5)	
N1	0.8123 (9)	0.3822 (8)	0.5717 (6)	0.0402 (19)	
N2	0.6167 (9)	0.4091 (8)	0.6379 (7)	0.048 (2)	
C1	1.1828 (11)	0.6965 (10)	0.8263 (9)	0.051 (3)	
H1A	1.2417	0.7576	0.7941	0.077*	
H1B	1.2489	0.6572	0.8691	0.077*	
H1C	1.1438	0.7442	0.8726	0.077*	
C2	0.6662 (11)	0.3300 (10)	0.5714 (8)	0.043 (2)	
C3	0.5668 (12)	0.2033 (10)	0.5068 (8)	0.053 (3)	
H3	0.4691	0.1650	0.5125	0.063*	
C4	0.6138 (14)	0.1358 (11)	0.4352 (9)	0.057 (3)	
H4	0.5499	0.0500	0.3922	0.069*	
C5	0.7594 (14)	0.1978 (11)	0.4276 (9)	0.057 (3)	
H5	0.7920	0.1568	0.3759	0.068*	
C6	0.8527 (12)	0.3189 (11)	0.4967 (8)	0.045 (2)	
H6	0.9502	0.3596	0.4913	0.054*	
C7	0.4833 (11)	0.3477 (11)	0.6713 (9)	0.054 (3)	
H7A	0.3919	0.3019	0.6060	0.064*	
H7B	0.4630	0.4184	0.7128	0.064*	
C8	0.5046 (12)	0.2502 (10)	0.7386 (8)	0.046 (3)	
C9	0.6496 (15)	0.2476 (13)	0.7969 (10)	0.066 (3)	
H9	0.7407	0.3058	0.7926	0.080*	
C10	0.656 (2)	0.1572 (17)	0.8612 (12)	0.090 (5)	
H10	0.7536	0.1558	0.8997	0.109*	
C11	0.527 (3)	0.0706 (16)	0.8707 (13)	0.098 (6)	
H11	0.5352	0.0092	0.9130	0.117*	
C12	0.385 (2)	0.0762 (14)	0.8161 (14)	0.090 (5)	
H12	0.2951	0.0215	0.8248	0.108*	
C13	0.3726 (14)	0.1606 (12)	0.7495 (11)	0.067 (4)	
H13	0.2742	0.1588	0.7101	0.080*	
C14	0.6497 (11)	0.5486 (10)	0.6317 (8)	0.042 (2)	
H14A	0.5702	0.5802	0.6461	0.051*	
H14B	0.6510	0.5567	0.5592	0.051*	
C15	0.7980 (11)	0.6491 (9)	0.8610 (8)	0.039 (2)	
C16	0.6740 (13)	0.6856 (12)	0.8728 (9)	0.058 (3)	
H16	0.6184	0.7164	0.8183	0.069*	
C17	0.6329 (15)	0.6761 (14)	0.9664 (11)	0.072 (4)	
H17	0.5494	0.7000	0.9733	0.086*	
C18	0.7140 (15)	0.6323 (12)	1.0477 (10)	0.063 (3)	
H18	0.6868	0.6271	1.1101	0.075*	
C19	0.8370 (15)	0.5957 (11)	1.0363 (9)	0.061 (3)	
H19	0.8923	0.5640	1.0904	0.073*	
C20	0.8774 (13)	0.6066 (10)	0.9431 (8)	0.048 (3)	
H20	0.9621	0.5840	0.9370	0.058*	
C21	0.8791 (11)	0.8172 (10)	0.7178 (9)	0.044 (2)	
C22	0.9235 (16)	0.9260 (11)	0.8040 (12)	0.075 (4)	
H22	0.9322	0.9134	0.8738	0.090*	
C23	0.955 (2)	1.0538 (16)	0.788 (2)	0.107 (6)	
H23	0.9901	1.1264	0.8481	0.129*	
C24	0.9375 (19)	1.0761 (16)	0.688 (2)	0.111 (7)	
H24	0.9531	1.1622	0.6779	0.133*	
C25	0.8956 (16)	0.9686 (19)	0.6016 (16)	0.095 (5)	
H25	0.8833	0.9826	0.5318	0.115*	
C26	0.8710 (13)	0.8394 (13)	0.6154 (10)	0.062 (3)	
H26	0.8493	0.7687	0.5566	0.074*	

### Atomic displacement parameters (Å^2^)

**Table:**

	*U*^11^	*U*^22^	*U*^33^	*U*^12^	*U*^13^	*U*^23^
Pt1	0.0336 (2)	0.0378 (2)	0.0384 (2)	0.01434 (16)	0.01476 (16)	0.01323 (16)
Cl1	0.0534 (16)	0.084 (2)	0.073 (2)	0.0448 (16)	0.0267 (15)	0.0198 (17)
P1	0.0353 (13)	0.0311 (13)	0.0347 (13)	0.0130 (10)	0.0094 (10)	0.0080 (10)
N1	0.046 (5)	0.037 (5)	0.041 (5)	0.014 (4)	0.017 (4)	0.016 (4)
N2	0.034 (4)	0.043 (5)	0.066 (6)	0.012 (4)	0.021 (4)	0.007 (4)
C1	0.044 (6)	0.042 (6)	0.058 (7)	0.007 (5)	0.003 (5)	0.027 (5)
C2	0.036 (5)	0.038 (6)	0.053 (6)	0.015 (5)	0.011 (5)	0.008 (5)
C3	0.048 (6)	0.042 (6)	0.052 (7)	0.008 (5)	0.012 (5)	−0.007 (5)
C4	0.067 (8)	0.037 (6)	0.059 (7)	0.007 (5)	0.024 (6)	0.005 (5)
C5	0.082 (8)	0.039 (6)	0.049 (7)	0.022 (6)	0.026 (6)	0.003 (5)
C6	0.056 (6)	0.054 (7)	0.043 (6)	0.031 (5)	0.031 (5)	0.015 (5)
C7	0.033 (5)	0.052 (7)	0.068 (7)	0.009 (5)	0.020 (5)	−0.001 (6)
C8	0.054 (7)	0.033 (6)	0.046 (6)	0.003 (5)	0.031 (5)	−0.009 (5)
C9	0.061 (8)	0.067 (9)	0.065 (8)	0.016 (7)	0.023 (6)	0.010 (7)
C10	0.119 (13)	0.102 (13)	0.071 (10)	0.055 (11)	0.039 (9)	0.034 (9)
C11	0.182 (19)	0.068 (11)	0.074 (11)	0.050 (12)	0.083 (13)	0.023 (8)
C12	0.111 (13)	0.050 (9)	0.106 (13)	−0.005 (8)	0.073 (11)	0.008 (8)
C13	0.059 (7)	0.052 (8)	0.080 (9)	0.000 (6)	0.045 (7)	−0.011 (7)
C14	0.038 (5)	0.046 (6)	0.048 (6)	0.026 (5)	0.010 (5)	0.011 (5)
C15	0.043 (5)	0.033 (5)	0.040 (5)	0.013 (4)	0.013 (4)	0.004 (4)
C16	0.051 (7)	0.072 (8)	0.054 (7)	0.033 (6)	0.015 (5)	0.007 (6)
C17	0.075 (9)	0.090 (10)	0.074 (9)	0.044 (8)	0.051 (7)	0.004 (8)
C18	0.093 (10)	0.054 (8)	0.058 (8)	0.030 (7)	0.049 (7)	0.012 (6)
C19	0.090 (9)	0.054 (8)	0.058 (7)	0.037 (7)	0.038 (7)	0.021 (6)
C20	0.059 (7)	0.046 (6)	0.046 (6)	0.028 (5)	0.023 (5)	0.003 (5)
C21	0.038 (5)	0.039 (6)	0.060 (7)	0.018 (5)	0.016 (5)	0.019 (5)
C22	0.096 (10)	0.028 (7)	0.107 (11)	0.012 (6)	0.058 (9)	0.012 (7)
C23	0.106 (13)	0.044 (9)	0.18 (2)	0.013 (8)	0.074 (13)	0.024 (11)
C24	0.088 (11)	0.042 (9)	0.23 (3)	0.026 (8)	0.081 (14)	0.056 (13)
C25	0.062 (9)	0.111 (14)	0.143 (15)	0.034 (9)	0.042 (9)	0.100 (13)
C26	0.052 (7)	0.064 (8)	0.070 (8)	0.019 (6)	0.016 (6)	0.030 (7)

### Geometric parameters (Å, °)

**Table:**

Pt1—C1	2.039 (10)	C10—H10	0.9300
Pt1—P1	2.178 (2)	C11—C12	1.37 (2)
Pt1—N1	2.219 (8)	C11—H11	0.9300
Pt1—Cl1	2.361 (3)	C12—C13	1.36 (2)
P1—C15	1.811 (10)	C12—H12	0.9300
P1—C21	1.818 (10)	C13—H13	0.9300
P1—C14	1.828 (10)	C14—H14A	0.9700
N1—C6	1.326 (12)	C14—H14B	0.9700
N1—C2	1.338 (12)	C15—C20	1.355 (13)
N2—C2	1.401 (12)	C15—C16	1.392 (13)
N2—C14	1.461 (12)	C16—C17	1.399 (16)
N2—C7	1.464 (13)	C16—H16	0.9300
C1—H1A	0.9600	C17—C18	1.364 (17)
C1—H1B	0.9600	C17—H17	0.9300
C1—H1C	0.9600	C18—C19	1.382 (15)
C2—C3	1.387 (13)	C18—H18	0.9300
C3—C4	1.357 (14)	C19—C20	1.392 (15)
C3—H3	0.9300	C19—H19	0.9300
C4—C5	1.389 (16)	C20—H20	0.9300
C4—H4	0.9300	C21—C22	1.372 (15)
C5—C6	1.352 (15)	C21—C26	1.385 (15)
C5—H5	0.9300	C22—C23	1.38 (2)
C6—H6	0.9300	C22—H22	0.9300
C7—C8	1.490 (15)	C23—C24	1.34 (2)
C7—H7A	0.9700	C23—H23	0.9300
C7—H7B	0.9700	C24—C25	1.37 (2)
C8—C9	1.391 (16)	C24—H24	0.9300
C8—C13	1.399 (15)	C25—C26	1.388 (19)
C9—C10	1.383 (19)	C25—H25	0.9300
C9—H9	0.9300	C26—H26	0.9300
C10—C11	1.36 (2)		
			
C1—Pt1—P1	90.3 (3)	C9—C10—H10	118.6
C1—Pt1—N1	176.2 (4)	C10—C11—C12	118.2 (15)
P1—Pt1—N1	93.5 (2)	C10—C11—H11	120.9
C1—Pt1—Cl1	86.8 (3)	C12—C11—H11	120.9
P1—Pt1—Cl1	176.99 (10)	C13—C12—C11	120.7 (14)
N1—Pt1—Cl1	89.5 (2)	C13—C12—H12	119.6
C15—P1—C21	105.9 (5)	C11—C12—H12	119.6
C15—P1—C14	102.0 (5)	C12—C13—C8	121.7 (13)
C21—P1—C14	104.8 (5)	C12—C13—H13	119.1
C15—P1—Pt1	119.0 (3)	C8—C13—H13	119.1
C21—P1—Pt1	117.2 (3)	N2—C14—P1	107.1 (6)
C14—P1—Pt1	106.0 (3)	N2—C14—H14A	110.3
C6—N1—C2	118.0 (9)	P1—C14—H14A	110.3
C6—N1—Pt1	117.7 (7)	N2—C14—H14B	110.3
C2—N1—Pt1	123.5 (7)	P1—C14—H14B	110.3
C2—N2—C14	116.7 (8)	H14A—C14—H14B	108.5
C2—N2—C7	120.7 (9)	C20—C15—C16	118.0 (9)
C14—N2—C7	117.0 (8)	C20—C15—P1	121.5 (7)
Pt1—C1—H1A	109.5	C16—C15—P1	120.3 (8)
Pt1—C1—H1B	109.5	C15—C16—C17	120.2 (11)
H1A—C1—H1B	109.5	C15—C16—H16	119.9
Pt1—C1—H1C	109.5	C17—C16—H16	119.9
H1A—C1—H1C	109.5	C18—C17—C16	120.8 (11)
H1B—C1—H1C	109.5	C18—C17—H17	119.6
N1—C2—C3	121.4 (9)	C16—C17—H17	119.6
N1—C2—N2	117.4 (9)	C17—C18—C19	119.3 (11)
C3—C2—N2	121.3 (9)	C17—C18—H18	120.4
C4—C3—C2	119.3 (10)	C19—C18—H18	120.4
C4—C3—H3	120.3	C18—C19—C20	119.3 (11)
C2—C3—H3	120.3	C18—C19—H19	120.3
C3—C4—C5	118.5 (11)	C20—C19—H19	120.3
C3—C4—H4	120.8	C15—C20—C19	122.4 (10)
C5—C4—H4	120.8	C15—C20—H20	118.8
C6—C5—C4	118.9 (10)	C19—C20—H20	118.8
C6—C5—H5	120.6	C22—C21—C26	118.4 (11)
C4—C5—H5	120.6	C22—C21—P1	122.4 (9)
N1—C6—C5	123.2 (10)	C26—C21—P1	119.2 (9)
N1—C6—H6	118.4	C21—C22—C23	120.5 (15)
C5—C6—H6	118.4	C21—C22—H22	119.7
N2—C7—C8	114.6 (9)	C23—C22—H22	119.7
N2—C7—H7A	108.6	C24—C23—C22	121.8 (18)
C8—C7—H7A	108.6	C24—C23—H23	119.1
N2—C7—H7B	108.6	C22—C23—H23	119.1
C8—C7—H7B	108.6	C23—C24—C25	118.1 (16)
H7A—C7—H7B	107.6	C23—C24—H24	120.9
C9—C8—C13	117.5 (12)	C25—C24—H24	120.9
C9—C8—C7	123.8 (10)	C24—C25—C26	121.7 (16)
C13—C8—C7	118.6 (11)	C24—C25—H25	119.2
C10—C9—C8	119.0 (13)	C26—C25—H25	119.2
C10—C9—H9	120.5	C21—C26—C25	119.2 (14)
C8—C9—H9	120.5	C21—C26—H26	120.4
C11—C10—C9	122.9 (16)	C25—C26—H26	120.4
C11—C10—H10	118.6		
			
C1—Pt1—P1—C15	−69.7 (5)	C10—C11—C12—C13	4(2)
N1—Pt1—P1—C15	110.5 (4)	C11—C12—C13—C8	−4(2)
Cl1—Pt1—P1—C15	−57 (2)	C9—C8—C13—C12	1.5 (16)
C1—Pt1—P1—C21	59.9 (5)	C7—C8—C13—C12	−175.0 (11)
N1—Pt1—P1—C21	−119.9 (4)	C2—N2—C14—P1	−90.2 (9)
Cl1—Pt1—P1—C21	73 (2)	C7—N2—C14—P1	115.7 (8)
C1—Pt1—P1—C14	176.3 (5)	C15—P1—C14—N2	−72.7 (7)
N1—Pt1—P1—C14	−3.5 (4)	C21—P1—C14—N2	177.1 (6)
Cl1—Pt1—P1—C14	−171 (2)	Pt1—P1—C14—N2	52.5 (7)
C1—Pt1—N1—C6	−22 (5)	C21—P1—C15—C20	−125.7 (9)
P1—Pt1—N1—C6	154.9 (7)	C14—P1—C15—C20	125.0 (9)
Cl1—Pt1—N1—C6	−25.8 (7)	Pt1—P1—C15—C20	8.8 (10)
C1—Pt1—N1—C2	148 (5)	C21—P1—C15—C16	58.9 (9)
P1—Pt1—N1—C2	−35.2 (8)	C14—P1—C15—C16	−50.5 (9)
Cl1—Pt1—N1—C2	144.2 (8)	Pt1—P1—C15—C16	−166.7 (7)
C6—N1—C2—C3	10.0 (15)	C20—C15—C16—C17	−1.0 (16)
Pt1—N1—C2—C3	−159.9 (8)	P1—C15—C16—C17	174.6 (10)
C6—N1—C2—N2	−169.3 (9)	C15—C16—C17—C18	1(2)
Pt1—N1—C2—N2	20.8 (13)	C16—C17—C18—C19	−1(2)
C14—N2—C2—N1	47.3 (12)	C17—C18—C19—C20	1.2 (18)
C7—N2—C2—N1	−159.6 (10)	C16—C15—C20—C19	1.6 (16)
C14—N2—C2—C3	−132.0 (10)	P1—C15—C20—C19	−174.0 (9)
C7—N2—C2—C3	21.1 (15)	C18—C19—C20—C15	−1.7 (18)
N1—C2—C3—C4	−6.0 (16)	C15—P1—C21—C22	21.9 (11)
N2—C2—C3—C4	173.3 (10)	C14—P1—C21—C22	129.3 (10)
C2—C3—C4—C5	−1.3 (17)	Pt1—P1—C21—C22	−113.6 (9)
C3—C4—C5—C6	4.3 (18)	C15—P1—C21—C26	−161.4 (8)
C2—N1—C6—C5	−7.0 (15)	C14—P1—C21—C26	−54.0 (9)
Pt1—N1—C6—C5	163.6 (8)	Pt1—P1—C21—C26	63.1 (9)
C4—C5—C6—N1	−0.2 (17)	C26—C21—C22—C23	1.6 (19)
C2—N2—C7—C8	61.9 (13)	P1—C21—C22—C23	178.3 (11)
C14—N2—C7—C8	−145.1 (9)	C21—C22—C23—C24	3(2)
N2—C7—C8—C9	20.8 (15)	C22—C23—C24—C25	−4(3)
N2—C7—C8—C13	−162.9 (9)	C23—C24—C25—C26	0(2)
C13—C8—C9—C10	0.5 (16)	C22—C21—C26—C25	−5.1 (16)
C7—C8—C9—C10	176.7 (11)	P1—C21—C26—C25	178.1 (9)
C8—C9—C10—C11	0(2)	C24—C25—C26—C21	4(2)
C9—C10—C11—C12	−2(2)		

### Hydrogen-bond geometry (Å, °)

**Table:**

Cg1 is the centroid of the C15–C20 benzene ring.

**Table:**

*D*—H···*A*	*D*—H	H···*A*	*D*···*A*	*D*—H···*A*
C23—H23···Cg1^i^	0.93	3.10	4.001 (2)	154
C7—H7B···Cl1^ii^	0.97	2.72	3.392 (2)	127

Symmetry codes: (i) −*x*+2, −*y*+2, −*z*+2; (ii) *x*−1, *y*, *z*.
